# Differential Phase Coupler Using Balun-Type Power Divider

**DOI:** 10.3390/mi16050496

**Published:** 2025-04-24

**Authors:** Chatrpol Pakasiri, Chung-Yu Chang, Sen Wang

**Affiliations:** 1 The College of Advanced Manufacturing Innovation, King Mongkut’s Institute of Technology Ladkrabang, Bangkok 10520, Thailand; 2 The Department of Electronic Engineering, National Taipei University of Technology, Taipei 10608, Taiwan; fighter0106@gmail.com

**Keywords:** differential phase coupler, common inductor, IPD process, lumped transmission line, three-port hybrid coupler, Wilkinson-type balun

## Abstract

This paper presents a differential phase coupler design methodology in the IPD process. The coupler consists of a balun and two phase-shifter circuits. The compact balun was designed with lumped components and a common inductor. Each output of the balun circuit was connected to a phase shifter with an opposite phase to make a desired output phase. In the design example, a three-port 90-degree hybrid coupler was implemented on the IPD process to operate at the 6 GHz WIFI frequency. The post-simulation showed that all reflection coefficients were below −19 dB, with an insertion loss of 1.76 dB and isolation of 20 dB. The core chip size was only 0.02λ_0_ × 0.018λ_0_.

## 1. Introduction

Couplers with differential phase output signals are widely used in many applications, e.g., in phase array antennas [1,2,3] and wireless communication systems [4]. In the design of couplers with differential phase output, a wide range of circuits and applications have been studied. The Wilkinson coupler is popular for in-phase output couplers that divide the input signal into two or more outputs. In order to make an opposite phase at the output, some modifications must be made in the circuit topology. In [5], Pakasiri et al. used a lumped circuit with a common inductor to make a Wilkinson-type balun in a sub-GHz circuit. Because of the small electrical length in the extremely high-frequency range (EHF), distributed transmission lines can be implemented on a chip. Gong et al. [6] and Hsu et al. [7] used distributed transmission lines in modified Wilkinson-type structures in the design of balun circuits.

The Marchand balun is a typical balun coupler circuit in distributed form. To implement it on-chip or in-package, the operating frequency is normally in the EHF region. Xiong et al. used through-silicon via distributed lines in implementing a compact 3D Marchand balun in the EHF range [8]. With the double-layer conductors with the through-silicon via, the circuit area was reduced.

Transformer-type baluns are also used widely [9,10,11,12]. The advantage of the transformer-type baluns is their small circuit size, and the transmission coefficients can be designed with very good signal coupling. Nevertheless, the matching at the ports is of concern since only a few components were used in the circuit, making it challenging to match the port impedance.

For 90-degree phase differences or hybrid couplers, the circuits can be three or four ports. Four-port directional couplers were implemented in a distributed form with arbitrary phase output [12,13] and on-chip using lumped components for RFID application [14]. The three-port hybrid couplers were designed in distributed forms [15,16,17,18] and in transformer-type structures [19]. In the implementation of the distributed forms, there are two main methods: (1) two cascaded circuits consisting of a power divider and phase shifter [15,16,17] and (2) a single circuit [18]. The advantages of the two cascaded circuits are (1) each circuit can be designed individually, and (2) the output phase can be arbitrarily chosen, while that of the single circuit is the smaller circuit area. Then, designing a phase shifter is a key part of arbitrary phase coupler circuits. For wideband couplers, wideband phase shifters are also required. Much research on passive phase shifters focused on wideband circuits using distributed forms [20] and lumped components [21,22,23,24].

In this work, an arbitrary phase three-port coupler on a chip was proposed with two cascaded circuits consisting of a balun and a phase shifter. The balun was chosen instead of the conventional in-phase power divider so that the output signals of the phase shifter circuits can be of equal magnitude and opposite phase, making it possible for a smaller-sized circuit. Also, since the electrical length of the phase shifter circuits on the chip using lumped transmission lines should be short so that it can perform closely to the distributed ones, the larger positive phase shifter can be avoided by replacing it with a negative one. The inductors used in the phase shifter circuit are then smaller because of the shorter electrical length, leading to a smaller circuit size. The choice of balun is then suitable to provide differential negative phase output. Overall, the system can provide differential phase outputs between 0° and −180°.

The coupler circuit was in lumped component form, so it was suitable for on-chip implementation working in the sub-GHz range. The balun circuit was in Wilkinson-type form [5,6,25], while the phase shifter was implemented using lumped left- and right-handed transmission lines [26]. As an example of the design methodology, a prototype 90° coupler circuit was implemented to work at a 6 GHz WIFI frequency. A common inductor was integrated into the balun design to make the chip area smaller. The phase shifter circuit was also modified to reduce the losses from the port impedance mismatch.

This paper is organized as follows. Section 2 introduced a Wilkinson-type balun with shorter length transmission lines. Section 3 described the differential phase coupler. Finally, Section 4 concluded this paper.

## 2. Wilkinson-Type Balun with Shorter Length Transmission Lines

**Figure 1:**
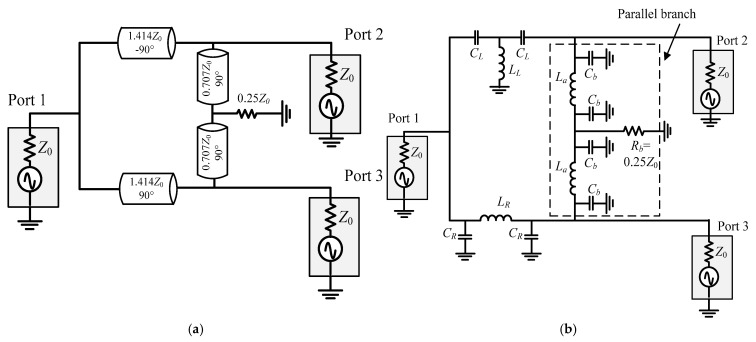
Schematic of the Wilkinson-type balun with (**a**) distributed transmission lines form and (**b**) lumped transmission lines form.

Wilkinson-type baluns used 90°/−90° transmission lines in the serial branches and distributed lines with a resistor placed between the output ports (in parallel branch), as shown in Figure 1a. The lumped transmission lines are usually used when it is implemented on-chip, as shown in Figure 1b. The negative phase transmission line is implemented by a left-handed transmission line, while the positive one is replaced by a right-handed transmission line [5]. In the lumped transmission lines, the inductor occupies the largest area of the circuit.

**Figure 2:**
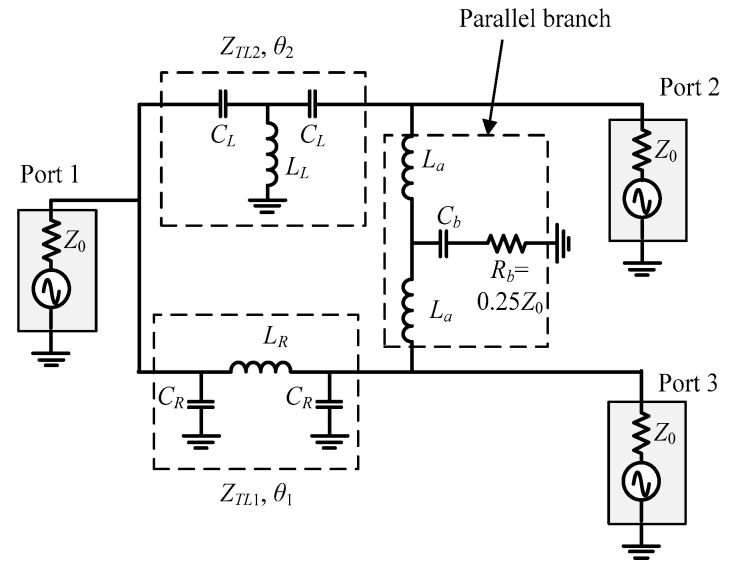
Schematic of the reduced-phase lumped-transmission lines with lumped components at the parallel branch.

To reduce the circuit size, two methods can be applied: (1) adjust the electrical length of the lumped-transmission line with a smaller inductor and/or (2) replace lumped transmission lines at the parallel branch with fewer components. In this paper, a new topology with these techniques was studied. The schematic is shown in Figure 2.

**Figure 3:**
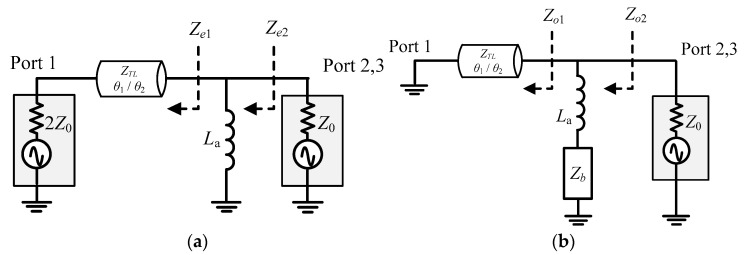
Schematics for the (**a**) even-mode and (**b**) odd-mode analysis.

The phases of lumped transmission lines in Figure 2 are related by

(1) $$\theta_{2}=\theta_{1}-180^{\circ }$$

where $\theta_{1}$ is the electrical length of the right-handed lumped-transmission line, while $\theta_{2}$ is the electrical length of the left-handed one. The characteristic impedance of the lumped transmission lines and the lumped components can be found by even- and odd-mode analysis, shown in Figure 3.

The characteristic impedance of the transmission lines and the line electrical lengths, $L_{a}$ and $Z_{b}=R_{b}+jX_{b}$, can be found from (2)–(5).

(2) $$Z_{0}=\frac{-\omega Z_{TL}^{2}L_{a}\tan(\theta )\left(2Z_{TL}Z_{0}-2\omega L_{a}Z_{0}\tan(\theta )\right)+2Z_{0}Z_{TL}\omega L_{a}\left(Z_{TL}^{2}\tan(\theta )+\omega L_{a}Z_{TL}\right)}{{\left(2Z_{TL}Z_{0}-2\omega L_{a}Z_{0}\tan(\theta )\right)}^{2}+{\left(Z_{TL}^{2}\tan(\theta )+\omega L_{a}Z_{TL}\right)}^{2}}$$

(3) $$Z_{TL}^{3}\frac{\tan^{2}(\theta )}{4Z_{0}^{2}}+Z_{TL}^{2}\frac{\omega L_{a}\tan(\theta )}{4Z_{0}^{2}}+Z_{TL}-\omega L_{a}\tan(\theta )=0$$

(4) $$Z_{0}\left(R_{b}^{2}+{\left(Z_{TL}\tan(\theta )+\omega L_{a}+X_{b}\right)}^{2}\right)-R_{b}Z_{TL}^{2}tan^{2}(\theta )=0$$

(5) $$\left(\omega L_{a}+X_{b}\right)Z_{TL}\tan(\theta )+{\left(\omega L_{a}+X_{b}\right)}^{2}+R_{b}^{2}=0$$

All components in the circuit are related to each other. Variation in some components affects the performance of the circuit. In the manufacturing process, capacitor values are quite stable. On the other hand, the inductors have some variations in both their values and losses. The inductor losses have a major impact on the impedance matching and bandwidth of the overall circuit. Choosing different electrical length lines implies choosing different inductor values in the design. In the following, the impact of the inductors on the bandwidth and phase variation is studied numerically with four different designs.

**Figure 4:**
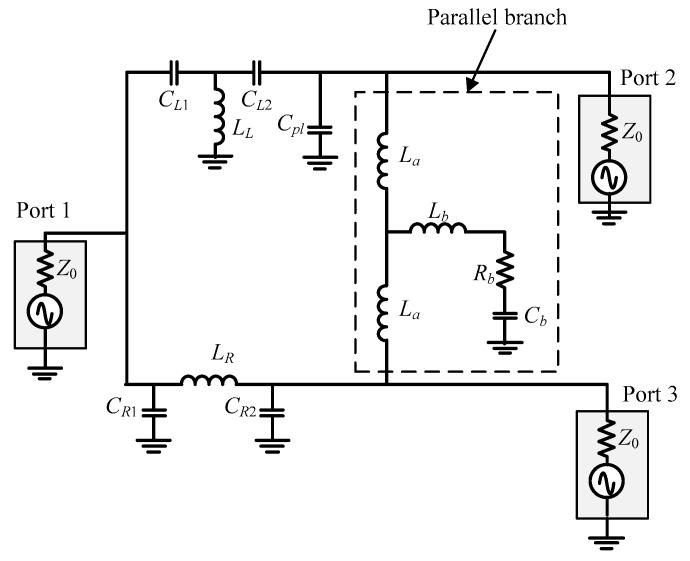
Schematics for the modified balun circuit [5].

The four different ideal lumped circuits operating at 6 GHz are shown in Table 1. The designs #1 with 60°/−120° and #2 with 75°/−105° are referred to in Figure 2. The design #3 with 90°/−90° using lumped transmission lines is referred to in Figure 1b, and the design #4 with 90°/−90° with a modified circuit is shown in Figure 4. Table 1 shows the design parameters with different *Q* values of the inductors. Their performances in terms of fractional bandwidth and maximum phase variation are summarized in Table 2. To identify the bandwidth, the −10 dB level for the reflection/isolation and 1.6 dB insertion loss were used.

In design #4, the two inductors, *L_a_*, in Figure 2, were replaced with a common inductor. The ideal circuit with the 90°/−90° lines in Figure 2 cannot be realized with lumped components since the even and odd modes require open and short impedances in the parallel branch. With some losses, the structure in Figure 2 could be used. Nevertheless, the size of the inductors for the 90°/−90° design needed was larger than the others. To further reduce the size of the inductor *L_a_* and improve the reflection losses, the circuit of design #4 was modified by replacing the inductors with a common inductor. The impedance matching in the even and odd modes can be easily controlled with the addition of the capacitor, *C_b_*, and the resistor, *R_b_*. A detailed analysis of the circuit can be found in [5].

It can be inferred from Table 2 that design #3 would have been one of the best performers among others if the inductors had been lossless. It had the smallest phase variation, but it had a major drawback of the smallest bandwidth with the *Q* value of 10. In addition, the exact parallel branch impedance was the key for the even and odd mode design methods. Design #3 needed the resonant effect in the even mode and impedance transformation of the lumped transmission lines in the parallel branch. Therefore, the component values needed to be precise to obtain the best performance. In practice, the implementation was difficult to accomplish because of many parasitic forms in the layout.

Generally, for the integrated passive device process, the quality factor of inductors was expected to be in the range of 15–20. Design #4 was chosen in implementing the arbitrary phase coupler so that the coupler would have a wider bandwidth with small phase variations. The matching impedance in the even- and odd-mode circuits with the layout parasitics and losses also makes the common inductor smaller. After the layout optimization, the design parameters at 6 GHz are *C_L_*_1_ = 0.394 pF, *C_L_*_2_ = 0.39 pF, *L_L_* = 1.876 nH, *C_pl_* = 0.65 pF, *C_R_*_1_ = 0.246 pF, *C_R_*_2_ = 1 pF, *L_R_* = 1.876 nH, *L*_a_ = 1 nH, *L_b_* = 0.385 nH, *C_b_* = 1.23 pF, and *R_b_* = 5.25 Ω. This balun was chosen as one of the building blocks for the differential phase coupler.

## 3. Differential Phase Coupler

**Figure 5:**
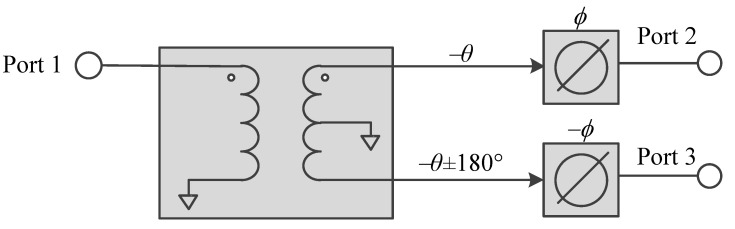
Diagram for the differential phase coupler.

The differential phase coupler consists of the balun and the phase shifters, as shown in Figure 5. Ideally, the phase shifters shown are lumped transmission lines with the same characteristic impedance as the port impedance and have the electrical length, *ϕ*. To achieve an output differential phase, ψ, the relationship between the output differential phase and the electrical length of the phase shifters, ϕ, is

(6) $$\psi =2\phi \pm 180^{\circ }$$

Initially, the balun from design #4 in Table 2 with *Q* = 10 was used to cascade with phase shifters. The phase of the phase shifters was chosen to be opposite to those of the transmission lines of the balun so that the output phase difference between phase shifter #1 and phase shifter #2 was minimum ($0{}^{\circ}\leq \left|\psi \right|\leq 180{}^{\circ}$).

**Figure 6:**
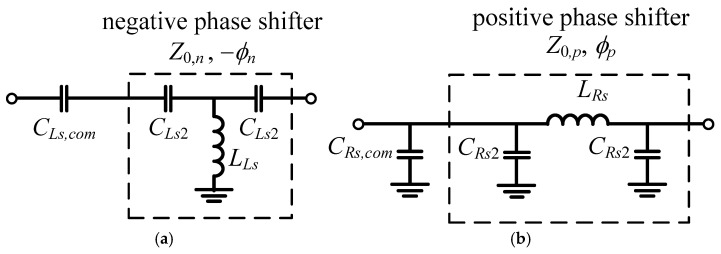
Schematics for (**a**) negative phase shifter and (**b**) positive phase shifter with compensation capacitors.

In implementation, the output impedances of the balun were not perfectly equal to the port impedance due to the parasitic inductance from the connecting line in the layout. Compensation capacitors can be added before the phase shifter circuits, as shown in Figure 6. The phase shifter circuits are implemented by lumped transmission line circuits with Figure 6a for negative phase shift and Figure 6b for positive phase shift. The characteristic impedance of the line and the electrical length are shown in (7)–(10), where $Z_{0,n}$ and $-\phi_{n}$ are the characteristic impedance and negative phase of the phase shifter, while $Z_{0,p}$ and $\phi_{p}$ are the characteristic impedance and positive phase of the phase shifters. In the design, the characteristic impedance of the lines is chosen to be the same as those of the port impedance.

(7) $$C_{LS2}=\frac{-\sin\left(-\phi_{n}\right)}{\omega Z_{0,n}\left(1-\cos(-\phi_{n})\right)}$$

(8) $$L_{LS}=\frac{-Z_{0,n}}{\omega \sin\left(-\phi_{n}\right)}$$

(9) $$C_{Rs2}=\frac{1}{\omega Z_{0,p}}\sqrt{\frac{1-\cos(\phi_{p})}{1+\cos(\phi_{p})}}$$

(10) $$L_{Rs}=\frac{Z_{0,p}}{\omega }\sqrt{1-\cos^{2}(\phi_{p})}$$

**Figure 7:**
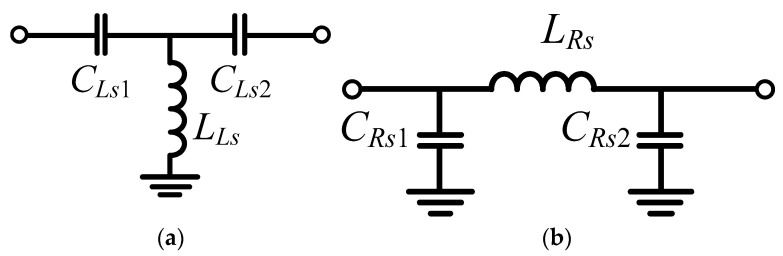
Schematics for (**a**) negative phase shifter and (**b**) positive phase shifter with phase correction.

In implementation, the compensation capacitors and the adjacent lumped transmission line capacitors are combined as shown in Figure 7. Their values are

(11) $$C_{Ls1}=\frac{C_{Ls,com}\cdot C_{Ls2}}{C_{Ls,com}+C_{Ls2}}$$

(12) $$C_{Rs1}=C_{Rs,com}+C_{Rs2}$$

Therefore, the phase shifters also function as an impedance transformer. The negative phase shifter in Figure 7a was used as the phase shifter #2, while the positive phase in Figure 7b was used as the phase shifter #1 in Figure 5.

**Figure 8:**
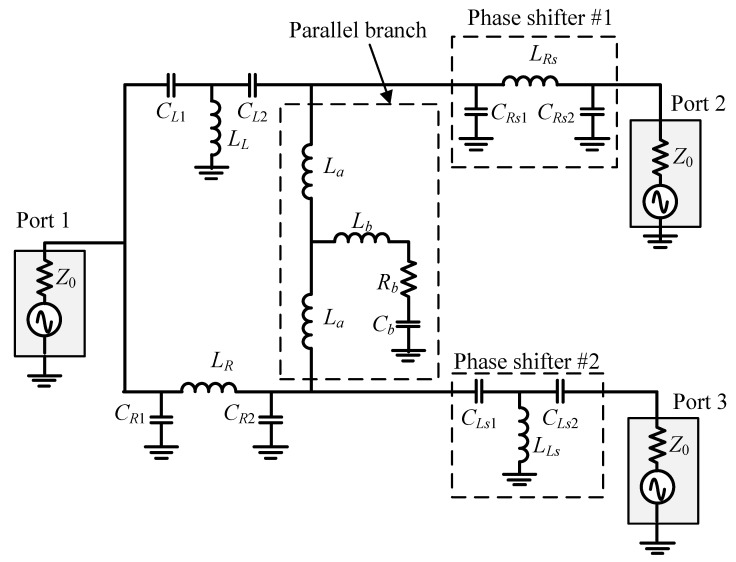
Schematics of the differential phase coupler.

The block circuits in Figure 5 were implemented as the circuit shown in Figure 8. To test the design method, a quadrature coupler operating at 6 GHz was designed, fabricated, and measured.

**Figure 9:**
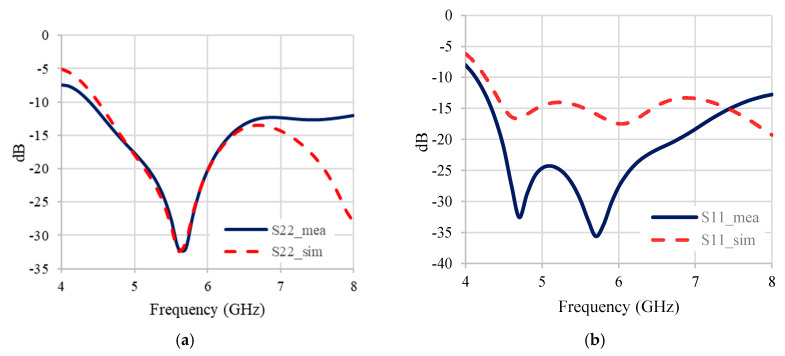

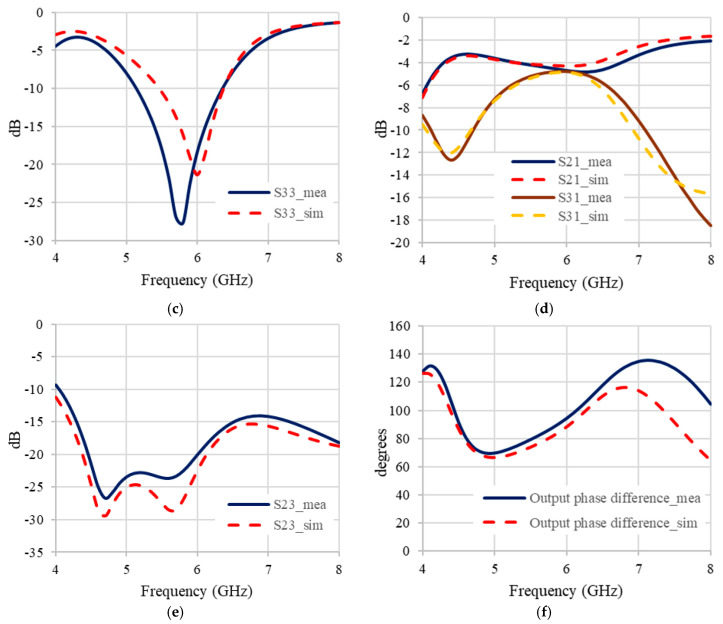
Results from layout simulation and measurement. (**a**) |S11| (**b**) |S22| (**c**) |S33| (**d**) |S21|, |S31| (**e**) |S23| (**f**) output phase difference.

The lumped components were adjusted to account for the layout parasitics. The design parameters at 6 GHz became *C_L_*_1_ = 1.53 pF, *C_L_*_2_ = 0.385 pF, *L_L_* = 1.635 nH, *C_R_*_1_ = 0.178 pF, *C_R_*_2_ = 0.98 pF, *L_R_* = 1.54 nH, *L_a_* = 0.81 nH, *L_b_* = 0.29 nH, *C_b_* = 1.7 pF, and *R_b_* = 7.2 Ω. The inductors were estimated to have *Q* ≈ 20. The resulting simulation and measurement are shown in Figure 9. In the figure, the measured data were added with ‘_mea’ in the legend, while the simulated data were added with ‘_sim’.

**Figure 10:**
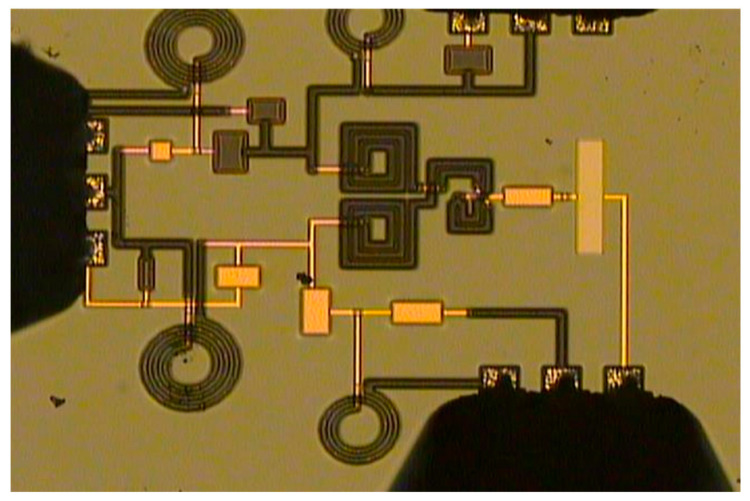
Chip photo. The core chip area is 989 μm × 893 μm $\left(0.02\lambda_{0}\times 0.018\lambda_{0}\right)$.

In Figure 9, the results from the measurement showed better matching at the input port than the simulated ones, while the measured reflection at the output ports was close to the simulated ones at the designed frequency. The transmission, the isolation, and the output phase difference were also in agreement. At the operating frequency, the measured results yielded reflection and isolation better than −19 dB, transmission better than −4.76 dB, and an output phase difference of 94.5°. Figure 10 shows the chip photo. The core size area was 989 μm × 893 μm. Table 3 shows the comparison between this work and others in the literature.

From the table, the designed hybrid coupler circuit of this work and [18] were implemented on-chip, while others were implemented on printed-circuit boards. The on-chip performance was expected to be inferior to those on PCB due to higher substrate loss. The reflection and isolation coefficients of this work were comparable with others, while the transmission coefficients were about 1 dB lower. The target phase difference was deviated by 4.5°. The chip size was only 0.02λ_0_ × 0.018λ_0_.

## 4. Conclusions

The three-port arbitrary phase coupler circuit on-chip was proposed in this paper. The circuit consists of a combination of balun and phase shifter circuits. Lumped-component Wilkinson-type baluns with lossy/lossless inductors were studied in this work. Three different transmission paths of the balun were also investigated. The ideal component simulations showed that the conventional transmission path length of 90°/−90° yielded the best performance. For layout implementation, a common inductor was used in the modified 90°/−90° circuit to reduce the size of the circuit. The phase shifter circuits were implemented by the modified right- and left-handed transmission lines. By choosing the line phase using the opposite signs to those of the transmission paths, the overall output phase difference could be kept minimal. With the proposed circuit, arbitrary phase outputs can be easily designed by adjusting the phase of the phase shifters.

An example circuit of a three-port 90° hybrid coupler was designed, fabricated, and measured. The measured chip showed a good performance compared with the others in the literature with a small size of $0.02\lambda_{0}\times 0.018\lambda_{0}$ in the IPD process.

## Figures and Tables

**Table 1:** Various balun designs for different length transmission lines.

No.	*Q*	*TL*_1_		*TL*_2_		*Parallel Branch*		
		*L_L_* (nH) (*R_L_*) (Ω)	*C_L_* (pF)	*L_R_* (nH) (*R_R_*) (Ω)	*C_R_* (pF)	*L_a_* (nH) (*R_a_*) (Ω)	*C_b_* (pF)	*R_b_* (Ω)
#1 60°/−120°	ideal	1.77	0.3	1.3	0.3	2.6	0.4	20
	20	1.77 (3.33)	0.3	1.3 (2.5)	0.3	2.6 (5)	0.4	20
	10	1.77 (6.66)	0.3	1.3 (5)	0.3	2.6 (10)	0.4	20
#2 75°/−105°	ideal	1.9	0.3	1.7	0.3	6.7	0.2	24
	20	1.9 (3.5)	0.3	1.7 (3.3)	0.3	6.7 (12.7)	0.2	24
	10	1.9 (7)	0.3	1.7 (6.6)	0.3	6.7 (25.4)	0.2	24
#3 90°/−90°	ideal	1.9	0.4	1.9	0.4	0.9	0.7	12.5
	20	1.9 (3.5)	0.4	1.9 (3.5)	0.4	0.9 (1.8)	0.7	12.5
	10	1.9 (7)	0.4	1.9 (7)	0.4	0.9 (3.5)	0.7	12.5
#4 90°/−90°	20	1.9 (3.5)	0.38	1.9 (3.5)	0.38	14 (26.4)	0.1	11.7
	10	1.9 (7)	0.38	1.8 (7)	0.38	15.7 (59)	0.1	33.4

**Table 2:** Simulation results of the balun designs.

No.	*Q*	Fractional Bandwidth (%)	Maximum Phase Deviation (°)
#1 60°/−120°	ideal	32.84	5.31
	20	33.58	6.35
	10	34.78	35.6
#2 75°/−105°	ideal	21.14	1.99
	20	24.39	8.12
	10	25.81	10.91
#3 90°/−90°	ideal	40	1.83
	20	33.9	4.07
	10	1.8	1.48
#4 90°/−90°	20	33.04	1.281
	10	21.85	1.516

**Table 3:** Various 90° hybrid coupler designs.

References	[15] *	[16] **	[17]	[18]	[19]	This Work
Process	PCB	PCB	PCB	PCB	CMOS	IPD
Type	Power divider and phase shifter	Power divider and phase shifter	Power divider and phase shifter	Combined power divider and phase shifter	Transformer	Balun and phase shifter
f0 (GHz)	3	0.65	1.75	2.5	26.5	6
S21, S31 (dB)	>−3.5	−3.2	>−5	>−4.68	−3.5	>−4.76
|S11|, |S22|, |S33| (dB)	<−16	−20	<−15	<−15	−19	<−18
|S23| (dB)	<−20	−25	<−55	<−20	N/A	<−20
Phase difference deviation (degrees)	<5	2	<3	<5	0.6	4.5
Size $\lambda_{0}^{2}$	0.239	0.047	N/A ***	0.35	1.4 × 10−4	3.6 × 10−4

* Estimated values from Figures 12–14 [15]. ** Estimated values from Figures 4 and 6 [16]. *** No data available [17].

## Data Availability

The original contributions presented in this study are included in the article. Further inquiries can be directed to the corresponding authors.
